# *Ahnak* deficiency attenuates high-fat diet-induced fatty liver in mice through FGF21 induction

**DOI:** 10.1038/s12276-021-00573-3

**Published:** 2021-03-30

**Authors:** Yo Na Kim, Jae Hoon Shin, Dong Soo Kyeong, Soo Young Cho, Mi-Young Kim, Hee Jung Lim, Maria Raquel Rojas Jimenez, Il Yong Kim, Mi-Ock Lee, Yun Soo Bae, Je Kyung Seong

**Affiliations:** 1 Laboratory of Developmental Biology and Genomics, Research Institute for Veterinary Science, and BK21 PLUS Program for Creative Veterinary Science Research, College of Veterinary Medicine, Seoul National University, Seoul, 08826 Korea; 2 Korea Mouse Phenotyping Center (KMPC), Seoul National University, Seoul, 08826 Korea; 3 Interdisciplinary Program for Bioinformatics, Program for Cancer Biology and BIO-MAX/N-Bio Institute, Seoul National University, Seoul, 08826 Korea; 4 National Cancer Center, Goyang-si, Gyeonggi-do 10408 Korea; 5 Department of Life Sciences, Ewha Womans University, Seoul, 03760 Korea; 6 Department of Pharmacy, College of Pharmacy and Bio-MAX Institute, Seoul National University, Seoul, 03760 Korea

**Keywords:** Mechanisms of disease, Metabolic syndrome

## Abstract

The AHNAK nucleoprotein has been determined to exert an anti-obesity effect in adipose tissue and further inhibit adipogenic differentiation. In this study, we examined the role of AHNAK in regulating hepatic lipid metabolism to prevent diet-induced fatty liver. *Ahnak* KO mice have reportedly exhibited reduced fat accumulation in the liver and decreased serum triglyceride (TG) levels when provided with either a normal chow diet or a high-fat diet (HFD). Gene expression profiling was used to identify novel factors that could be modulated by genetic manipulation of the *Ahnak* gene. The results revealed that fibroblast growth factor 21 (FGF21) was markedly increased in the livers of *Ahnak* KO mice compared with WT mice fed a HFD. *Ahnak* knockdown in hepatocytes reportedly prevented excessive lipid accumulation induced by palmitate treatment and was associated with increased secretion of FGF21 and the expression of genes involved in fatty acid oxidation, which are primarily downstream of PPARα. These results indicate that pronounced obesity and hepatic steatosis are attenuated in HFD-fed *Ahnak* KO mice. This may be attributed, in part, to the induction of FGF21 and regulation of lipid metabolism, which are considered to be involved in increased fatty acid oxidation and reduced lipogenesis in the liver. These findings suggest that targeting AHNAK may have beneficial implications in preventing or treating hepatic steatosis.

## Liver disease: A potential treatment for nonalcoholic fatty liver

A protein that modulates lipid accumulation could be a target for controlling nonalcoholic fatty liver disease (NAFLD). The AHNAK protein regulates fat cell development, and mice lacking AHNAK are less susceptible to obesity after being fed a HFD. South Korean researchers led by Je Kyung Seong of Seoul National University and Yun Soo Bae of Ewha Womans University, Seoul, have identified a mechanism by which AHNAK inhibition prevents lipid accumulation in NAFLD. The authors profiled gene expression in AHNAK-deficient mice, and noted a striking increase in levels of a signaling protein called FGF21 in the liver. This elevated FGF21 was in turn linked to increased breakdown and reduced production of fatty acids. Therapeutic interventions that inhibit AHNAK could help control NAFLD, a hard-to-treat condition that can ultimately lead to cirrhosis and liver failure.

## Introduction

Nonalcoholic fatty liver disease (NAFLD) has been identified as a common disorder that is characterized by increased hepatic triglyceride (TG) accumulation. NAFLD represents a broad spectrum of liver diseases ranging from nonalcoholic simple fatty liver to nonalcoholic steatohepatitis (NASH), hepatic fibrosis, and cirrhosis^1^. Hepatic lipid accumulation is known to be regulated by lipid uptake, de novo synthesis, oxidation, and transport of fatty acids to the circulation^2^. An imbalance in these complex metabolic processes can cause an excessive amount of hepatic TG accumulation.

Fibroblast growth factor 21 (FGF21) is a member of the fibroblast growth factor family consisting of 22 members that have diverse functions in the regulation of physiological homeostasis in metabolic tissues^3^. FGF21 is primarily synthesized and secreted from the liver^4^, and the action of circulating FGF21 is mediated through FGF receptors complexed with β-Klotho^5^. The expression of *Fgf21* is then induced by the activation of peroxisome proliferator-activated receptor alpha (PPARα). Nonesterified fatty acids bind to and activate PPARα. Ligand-bound PPARα then forms a heterodimer with retinoid X receptors to induce the expression of *Fgf21*. In cultured adipocytes, FGF21 signaling is transduced by activating the β-Klotho-FGFR1c complex. However, FGF21 signaling can be transduced in β-Klotho knockout mice, indicating the existence of a β-Klotho-independent FGF21 signaling pathway^6^. FGF21 has been known to act on adipose tissue to reduce plasma glucose and TGs, which in turn decreases body weight^7^. FGF21 is a potent regulator of adiponectin secretion in white adipose tissue and exerts body weight-lowering effects in brown adipose tissues and beige cells mediated by the induction of thermogenesis^8,9^. FGF21 also exerts direct effects on pancreatic islet cells to increase beta-cell function, survival^10^, and growth plate chondrocytes^11^. Treatment with FGF21 can reduce hepatic TG accumulation and hepatic steatosis, thus attenuating body weight gain in rodents and primates^12,13^. Additionally, FGF21 regulates energy homeostasis in adipocytes through an AMP-activated protein kinase (AMPK)/sirtuin 1 (SIRT1)/peroxisome proliferator-activated receptor gamma coactivator 1 alpha (PGC1α) signaling cascade, resulting in enhanced mitochondrial oxidative capacity^14–16^. Thus, FGF21 may represent a novel therapeutic molecule based on the findings that it protects animals from diet-induced obesity and reduces hepatic lipid accumulation, enhances glucose metabolism, and thus prevents hepatic steatosis, fibrosis, and NAFLD when administered to diabetic rodents^17,18^.

AHNAK is a very large protein that acts not only on calcium signaling or ion channels but also on nucleoproteins that are known to regulate a wide variety of biological functions^19^, such as adipogenesis^20^, browning^21^, tumor development^22^, and adipocyte differentiation^23^. In a previous study, we found that *Ahnak* KO mice exhibit strong resistance to high-fat diet (HFD)-induced obesity. Changes in the pattern of urinary metabolites in HFD-fed *Ahnak* KO mice suggest that the strong resistance to HFD-induced obesity in *Ahnak* KO mice is related to perturbations in amino acids that are related to fat metabolism^24^. Although *Ahnak* KO mice showed strong resistance to diet-induced obesity and hepatic steatosis, the role of AHNAK in the regulation of hepatic steatosis has not been studied.

In the present study, the role of AHNAK in diet-induced fatty liver disease was determined by using *Ahnak* KO mice. *Ahnak* KO mice have been shown to not have excessive hepatic lipid accumulation when fed a HFD. Gene expression analysis showed that a HFD upregulated *Fgf21* in the liver of *Ahnak* KO mice but not in WT littermates. Increased hepatic and circulating levels of FGF21 in HFD-fed *Ahnak* KO mice were associated with increased hepatic expression of genes involved in fatty acid oxidation and decreased expression of genes involved in lipogenesis compared with those in the WT littermates. Primary hepatocytes from *Ahnak* KO mice consistently exhibited decreased lipid accumulation and were accompanied by increased FGF21 secretion and cellular FGF21 protein levels. In addition, to enhance FGF21 production and secretion, *Ahnak*-deficient hepatocytes also exhibited increased expression of PPARα protein and its target genes in response to palmitate treatment. Characterization of phenotypic changes in *Ahnak* KO mice, such as reduced hepatic steatosis, decreased adiposity, and increased energy expenditure, establishes AHNAK as a novel regulator of FGF21 in modulating hepatic fatty acid metabolism. Thus, the present study establishes the role of AHNAK in the regulation of hepatocyte lipid metabolism through PPARα/FGF21 signaling.

## Materials and Methods

### Experimental animals

*Ahnak* KO mice were generated by disrupting exon 5 of the *Ahnak* gene as described^16^. *Ahnak* KO mice were obtained by crossing heterozygous breeders. Eight-week-old male KO and wild-type mice were randomly assigned and fed either regular chow or a 60% HFD (D12492; Research Diets Inc., NJ, USA) for 7 weeks. All animals were maintained at 24 ± 2 °C with 12 h of light per day and had free access to water in a specific pathogen-free barrier facility. These procedures were reviewed according to the “Guide for Animal Experiments” (edited by the Korean Academy of Medical Sciences) by the Institutional Animal Care and Use Committee at Seoul National University. The animal protocol was approved by the committee on the Ethics of Animal Experiments at Seoul National University (Permit Number: SNU-131024-6). All of the experiments were conducted to minimize the number of animals used^25^.

### Measurement of metabolic parameters in mice

A comprehensive animal metabolic monitoring system (CLAMS; Columbus Instruments, Columbus, OH) was used to evaluate the activity, food consumption, and energy expenditure of 8-week-old male KO and wild-type mice by switching the feeding method, such as feeding an NCD for 2 days, followed by a HFD for 4 days. Energy expenditure and food intake data were normalized with respect to lean body weight. The energy expenditure and respiratory exchange ratio (RER) were then calculated from gas exchange data [energy expenditure = (3.815 + 1.232 × RER) × VO_2_]. The RER was determined by the ratio of VCO_2_ to VO_2_, which changes depending on the energy source of the animal. When carbohydrates are the only substrate being oxidized, the RQ will be 1.0, whereas it will be 0.7 when only the fatty acids are oxidized. Activity was measured on the x- and z-axes using infrared beams to count the number of beam breaks during a specified measurement period. Feeding was measured by recording the difference in the scale measurement of the center feeder from one time point to another^26^.

### Histochemical study

For histological examination, mouse liver tissues were dissected and fixed in 10% neutral buffered formalin. The samples were subsequently embedded in paraffin or OCT compound. For hematoxylin and eosin (H&E) staining, tissue sections were cut at a thickness of 4 µm and were then stained using a commercial kit (HHS123, Sigma-Aldrich, St. Louis, MO). For Oil Red O staining, frozen samples were sectioned at 5 µm in thickness using a cryostat^27^. These sections were placed onto slides, washed with 60% isopropanol, and then stained with Oil Red O for 30 min.

### Measurement of VLDL secretion and lipid clearance rates

To assess VLDL secretion, serum triacylglycerol (TG) was measured in blood obtained from 4-hour-fasted mice for the indicated times after i.p. injection of Poloxamer 407 (1 mg/g of body weight) solution in PBS^28^. For measurement of lipid clearance rates, mice were fasted for 12 h and administered olive oil (Sigma-Aldrich, St. Louis, MO) by oral gavage. Blood was collected from the tail vein prior to gavage and 1, 2, 3, and 6 h after treatment. TGs were measured enzymatically with TG reagent (BioVision, Korea).

### Primary hepatocyte and HepG2 cell culture

Primary hepatocytes from WT and *Ahnak* KO mice were isolated using a two-step perfusion technique and cultured as described previously^29^. After cell attachment, hepatocytes were serum-starved for 4 h and then treated with 250 μM palmitate for 12 h. In some experiments, hepatocytes were treated with AICAR for the indicated time periods. HepG2 cells were purchased from the American Type Culture Collection and maintained in Dulbecco’s modified Eagle’s medium (DMEM; Gibco-Life Technologies, Grand Island, NY) containing high glucose, 10% FBS, 100 U/ml penicillin, and 100 mg/ml streptomycin, as described previously^30^. Control siRNA and siRNA for the *Ahnak* gene (Bioneer, Korea) were used for gene silencing. A cell steatosis model was established by culturing HepG2 cells in DMEM with BSA-conjugated palmitate (250 μM) for 12 or 24 h.

### Quantitative real-time PCR

Total RNA was extracted from the liver using the Total RNA Purification System (Invitrogen, Carlsbad, CA) according to the manufacturer’s protocol. Messenger RNA was reverse-transcribed using AccuPower CycleScript RT PreMix (Bioneer, Daejeon, Korea). Quantitative real-time PCR was performed with SYBR Green dye using the StepOnePlus™ Real-Time PCR System (Applied Biosystems, Cheshire, U.K.). Expression of the respective genes was normalized to the 36B4 signal as an internal control, and relative gene expression was quantitated by the comparative Ct method (ΔΔCt). The primer sequences of target genes are listed in Supplementary Table 1.

### Western blot analysis

Total protein was extracted from the liver of each group using protein lysis buffer (Invitrogen, Carlsbad, CA) containing phosphatase inhibitor (GenDEPOT, Barker, TX, USA). Protein extracts were resolved by 10% or 12% SDS-PAGE gels and were later transferred onto PVDF membranes (Millipore, Billerica, MA). The protein bands were detected with antibodies against pAMPK (Thr172), AMPK, pACC, ACC, PPARα, SCD1, CPT1 (Abcam, Cambridge, UK), and GAPDH (Cell Signaling Technologies, Beverly, MA) using the appropriate secondary HRP-conjugated antibodies (Cell Signaling Technologies, Beverly, MA). The blots were developed and imaged using the MicroChemi 4.2 system (DNR Bio-imaging system, Israel).

### FGF21 measurement

FGF21 concentrations in plasma and culture medium were measured by ELISA (R&D Systems, Minneapolis, MN) according to the manufacturer’s protocol. To measure FGF21 secretion, the hepatocytes were serum-starved for 4 h, and then the supernatants were collected and preserved in a −80 °C freezer until further analysis.

### Statistical analysis

The results are expressed as the means ± SEM. Student’s *t* tests were used to analyze gene expression differences between WT and *Ahnak* KO mice as measured by quantitative real-time PCR. Differences were considered significant at *p* < 0.05.

## Results

### *Ahnak* KO mice are resistant to HFD-induced hepatic steatosis

**Fig. 1:**
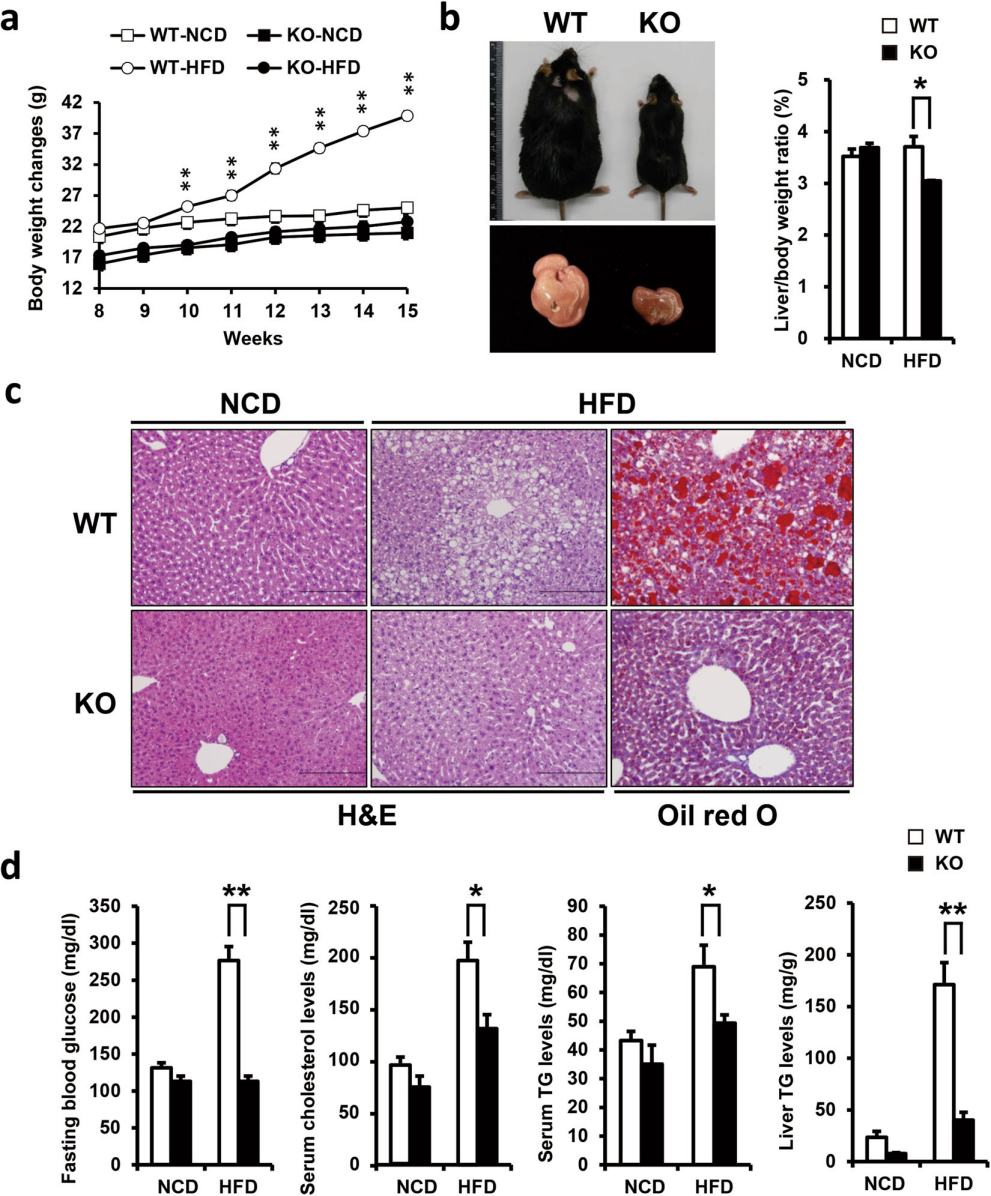
*Ahnak* KO mice are protected against diet-induced obesity and hepatic steatosis. **a** Body weight changes in mice receiving a 60% high-fat diet for 7 weeks. **b** Macroscopic appearance of the liver and mice. The liver-to-body weight ratio was measured. **c** H&E staining of WT and KO mice. Oil Red O staining of lipid droplets. Original magnification, x200. **d** Fasting blood glucose levels, serum cholesterol, and serum and hepatic TG contents were measured. Data were presented as the means ± SEM. *p < 0.05; **p < 0.01.

To determine the potential correlation between AHNAK expression and hepatic steatosis, we examined AHNAK expression in the livers of diet-induced and genetically induced obese mice. *Ahnak* mRNA was found to be significantly increased in the livers of both obese mouse models (Supplementary Fig. 1). To test the function of AHNAK in diet-induced hepatic steatosis, *Ahnak* KO mice and their wild-type littermates were fed a HFD containing 60% fat or a NCD. When 8-week-old male mice were fed a HFD for 7 weeks, *Ahnak* KO mice weighed less than WT mice in both the NCD and HFD groups. In addition, HFD-KO mice gained significantly less weight than WT mice (Fig. 1a, b).

In a previous study, we reported that the differences in body weight caused by fewer fat pads are due to impaired adipogenesis in KO mice^20^. Nevertheless, since the liver is a secondary fat storage site and an essential metabolic organ, subsequent studies have been conducted. The ratio of liver-to-body weight was observed to be significantly decreased in KO mice compared with WT mice fed a HFD (Fig. 1b). Histological analysis revealed that WT mice receiving a HFD exhibited severe hepatic steatosis with an accumulation of intracellular lipid droplets, whereas *Ahnak* KO mice did not display fatty liver symptoms (Fig. 1c). Consistent with the histological results, both hepatic and serum levels of TG decreased in the livers of HFD-fed *Ahnak* KO mice compared with WT mice. The basal level of blood glucose was also determined to decrease in the HFD-fed *Ahnak* KO mice compared with HFD-fed WT mice (Fig. 1d). The HFD-fed WT mice had elevated blood glucose levels, whereas HFD-fed *Ahnak* KO mice did not exhibit hyperglycemia. Total cholesterol levels were also reduced in HFD-fed *Ahnak* KO mice compared with WT mice (Fig. 1d). Overall, these observations indicate that *Ahnak* KO mice were significantly protected from HFD-induced obesity and hepatic steatosis.

### *Ahnak*-deficient mice exhibit decreased serum lipid clearance but not VLDL secretion

**Fig. 2:**
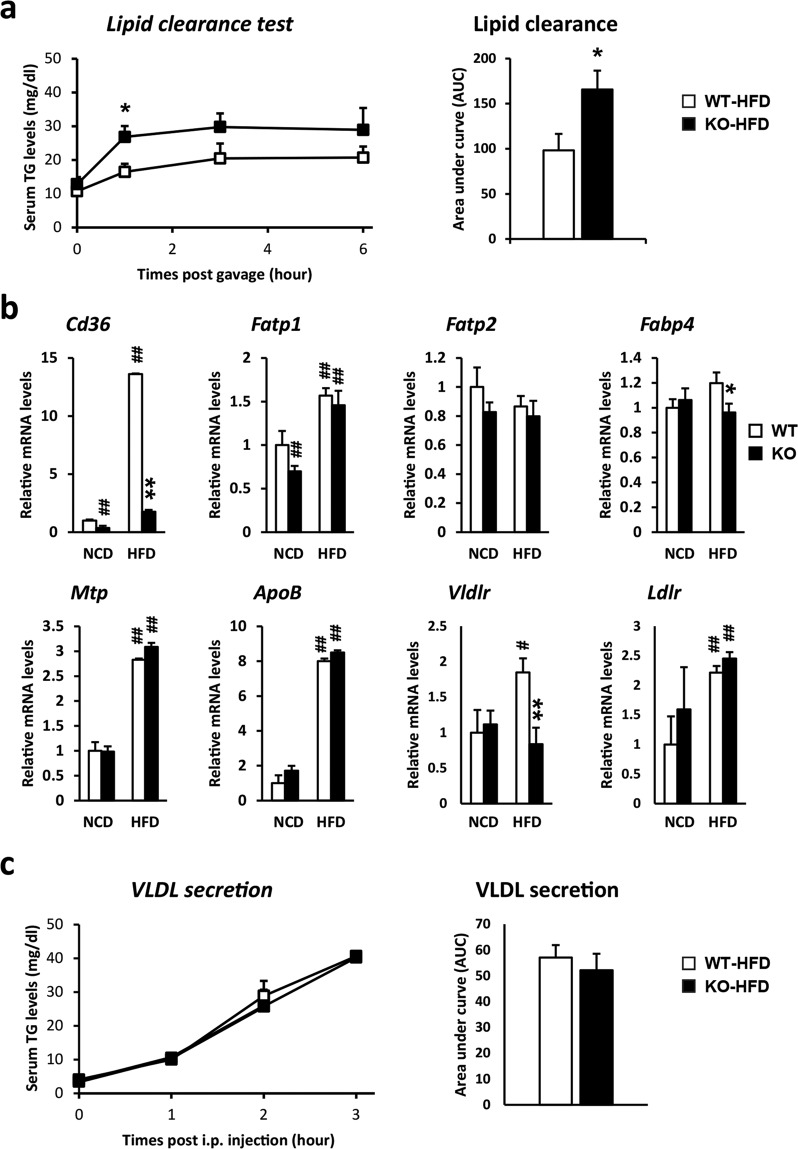
HFD-fed *Ahnak*-deficient mice have attenuated lipid clearance but not VLDL secretion. **a** Lipid clearance was estimated by measuring blood TG levels after oral olive oil administration. **b** Analysis of gene expression involved in VLDL secretion and lipid uptake after a 7-week HFD feeding by quantitative real-time PCR normalized to *36B4* (*n* = 4–6 mice). **c** VLDL secretion. Mice were treated with Poloxamer 407 to block VLDL clearance, and VLDL secretion was then estimated by measuring blood TG levels after Poloxamer 407 treatment. Data were presented as the means ± SEM. *p < 0.05; **p < 0.01.

To test whether lipid transport in the liver was altered in *Ahnak* KO mice, olive oil was administered to WT and KO mice, and the lipid clearance rate was evaluated. *Ahnak* KO mice were found to exhibit higher levels of serum TG than WT mice after olive oil treatment in the HFD group (Fig. 2a). However, there were no significant differences in serum TG levels between the WT and KO mice in the NCD group (Supplementary Fig. 2). To determine which molecular mechanism affects lipid transport in *Ahnak* KO mice, the mRNA expression of genes involved in lipid uptake was measured. The expression of *Cd36*, a membrane protein that facilitates the uptake of fatty acids and cholesterol, was markedly reduced in the liver of *Ahnak* KO mice fed either a NCD or a HFD, whereas the expression of fatty acid binding proteins involved in fatty acid uptake, such as *Fatp1*, *Fatp2*, and *Fabp4*, was similar or slightly altered between WT and KO mice (Fig. 2b). The current data suggest that *Ahnak* KO mice have reduced expression of genes related to fatty acid transport, which could explain the observed impaired lipid transport to the liver and reduced hepatic lipid accumulation (Fig. 1c). Additionally, to examine the effect of *Ahnak* deficiency on VLDL secretion, the animals were treated with Poloxamer 407, which inhibits lipoprotein lipase activity and VLDL secretion. The VLDL secretion rate was estimated by measuring the serum TG levels at each time point following Poloxamer 407 treatment. The VLDL secretion rate was similar between WT and KO mice fed either a NCD (Supplementary Fig. 2) or a HFD (Fig. 2c). Consistent with the VLDL secretion results, the expression levels of *ApoB* and *Mtp*, which promote VLDL assembly and secretion, were also similar between WT and KO mice (Fig. 2b).

### *Ahnak* deficiency in mice increases the production and secretion of hepatic FGF21

**Fig. 3:**
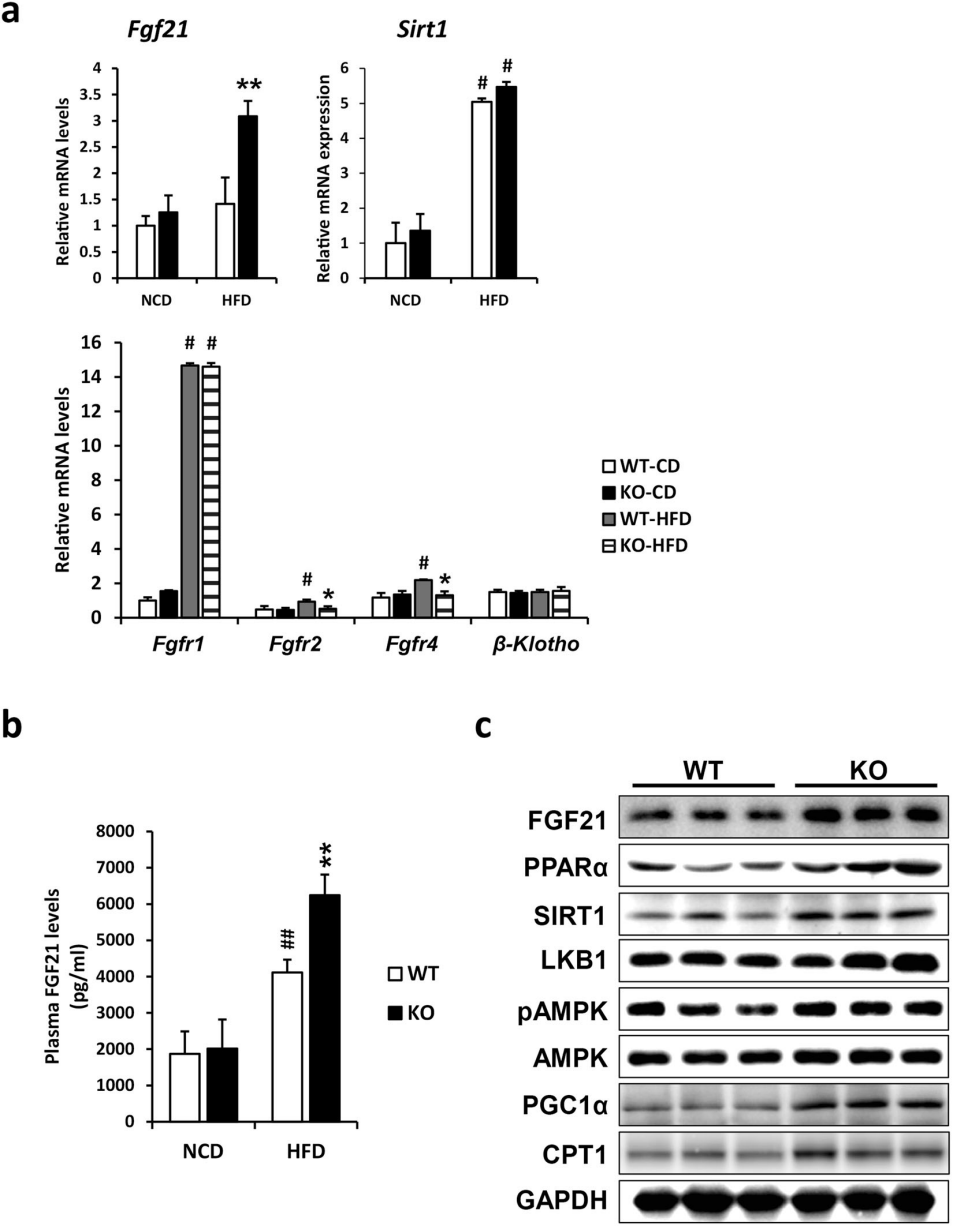
Loss of *Ahnak* in mice increases the production and secretion of FGF21. Measurement of selected inflammatory gene expression in the liver after a 7-week HFD feeding by quantitative real-time PCR and normalization to *36B4*. **a** Analysis of FGF21 expression and its receptors (*Fgfr1*, *Fgfr2*, *Fgfr4*, and coreceptor *β-Klotho*). **b** Plasma levels of FGF21 in *Ahnak* KO and WT mice fed chow and a HFD for 7 weeks. **c** Immunoblots showing the protein expression levels of FGF21 and PPARα and their downstream targets in HFD-fed *Ahnak* KO and WT mice. Data were presented as the means ± SEM (*n* = 4–6 mice). *p < 0.05; **p < 0.01.

As part of a screening to identify novel secreted proteins that could be modulated by *AHNAK* genetic manipulation, gene expression profiling revealed that *Fgf21* was increased by ~3.2-fold in the livers of *Ahnak* KO mice compared with those of WT mice under HFD conditions (accession No. GSE70119). Hierarchical clustering revealed that gene expression in the livers of WT mice that were fed a NCD was closely aligned with that of *Ahnak* KO mice. The livers of HFD-fed *Ahnak* KO mice had a similar cluster of genes compared with that of the NCD-fed group, whereas the livers of HFD-fed WT mice showed a distinctive differential expression gene (DEG) profile (Supplementary Fig. 3a). A gene ontology analysis of the DEGs revealed that *Ahnak* deficiency induced several metabolic pathways (Supplementary Fig. 3b). Downregulated genes in the livers of HFD-fed *Ahnak* KO mice were associated with the immune response. Genes involved in proinflammatory signaling were significantly downregulated in the livers of *Ahnak* KO mice fed a HFD, including chemokine (C-C motif) ligand 2 (*Ccl2*), chemokine (C-C motif) ligand 3 (*Ccl3*), tumor necrosis factor α (*Tnfα*), and *F4/80*. However, the expression levels of anti-inflammatory genes were not changed (Supplementary Fig. 3c). As expected, hepatic mRNA expression and protein levels of FGF21 also increased in *Ahnak* KO mice compared with WT mice fed a HFD (Fig. 3a). However, no difference was determined in *Fgf21* expression when the animals were fed a NCD. FGF21 activity is mediated through FGF receptors that interact with β-Klotho. A genetic deficiency of *Ahnak* was determined to have no effect on the hepatic expression of FGF receptors or β-Klotho when fed either a NCD or a HFD. Interestingly, FGFR1 expression was dramatically increased in the livers of both WT and *Ahnak* KO mice fed a HFD (Fig. 3a). These results indicate that HFD-induced circulating FGF21 acts primarily through binding to FGFR1. FGF21 is a significant metabolic regulator in several diabetic animal models. FGF21 can protect animals from diet-induced obesity and reduce hepatic lipid accumulation, enhance glucose metabolism, and thus prevent hepatic steatosis, fibrosis, and NAFLD when administered to diabetic rodents^8–10,12,15,16^. To elucidate the role of AHNAK in preventing diet-induced hepatic steatosis, we determined whether *Ahnak* deficiency regulates HFD-induced production and release of hepatic FGF21. As shown in Fig. 3c, the protein expression of hepatic FGF21 was found to be significantly increased in the livers of HFD-fed *Ahnak* KO mice. Furthermore, plasma FGF21 and PPARα protein levels were also elevated in *Ahnak* KO mice receiving a HFD (Fig. 3b, c). AMPK, SIRT1, and PGC1α have been identified as important regulators of mitochondrial biogenesis and function^31,32^, and LKB1, a serine threonine kinase, directly activates AMPK activity^33^. In addition, FGF21 regulates energy metabolism by activating the AMPK-SIRT1-PGC1α pathway^14,16^. Because the expression of FGF21 was increased in the livers of HFD-fed *Ahnak* KO mice, we determined whether the expression levels of these molecules were altered. Consistent with our hypothesis, we observed increased protein levels of LKB1, SIRT1, and PGC1α and increased amounts of phosphorylated AMPK. In addition, the rate-limiting enzyme of fatty acid oxidation, carnitine palmitoyltransferase 1 (CPT1), was upregulated (Fig. 3c). Despite increased expression of PGC1α, no differences were observed between the HFD-fed WT and *Ahnak* KO mice in the levels of mitochondrial oxidative phosphorylation complex proteins (Supplementary Fig. 4). These results indicate that pronounced obesity and hepatic steatosis are attenuated in *Ahnak* KO mice fed a HFD. This may be attributed, in part, to increased expression of genes associated with fatty acid oxidation and induction of FGF21 in the liver.

### *Ahnak* KO mice exhibit altered hepatic expression of key genes involved in lipid metabolism

**Fig. 4:**
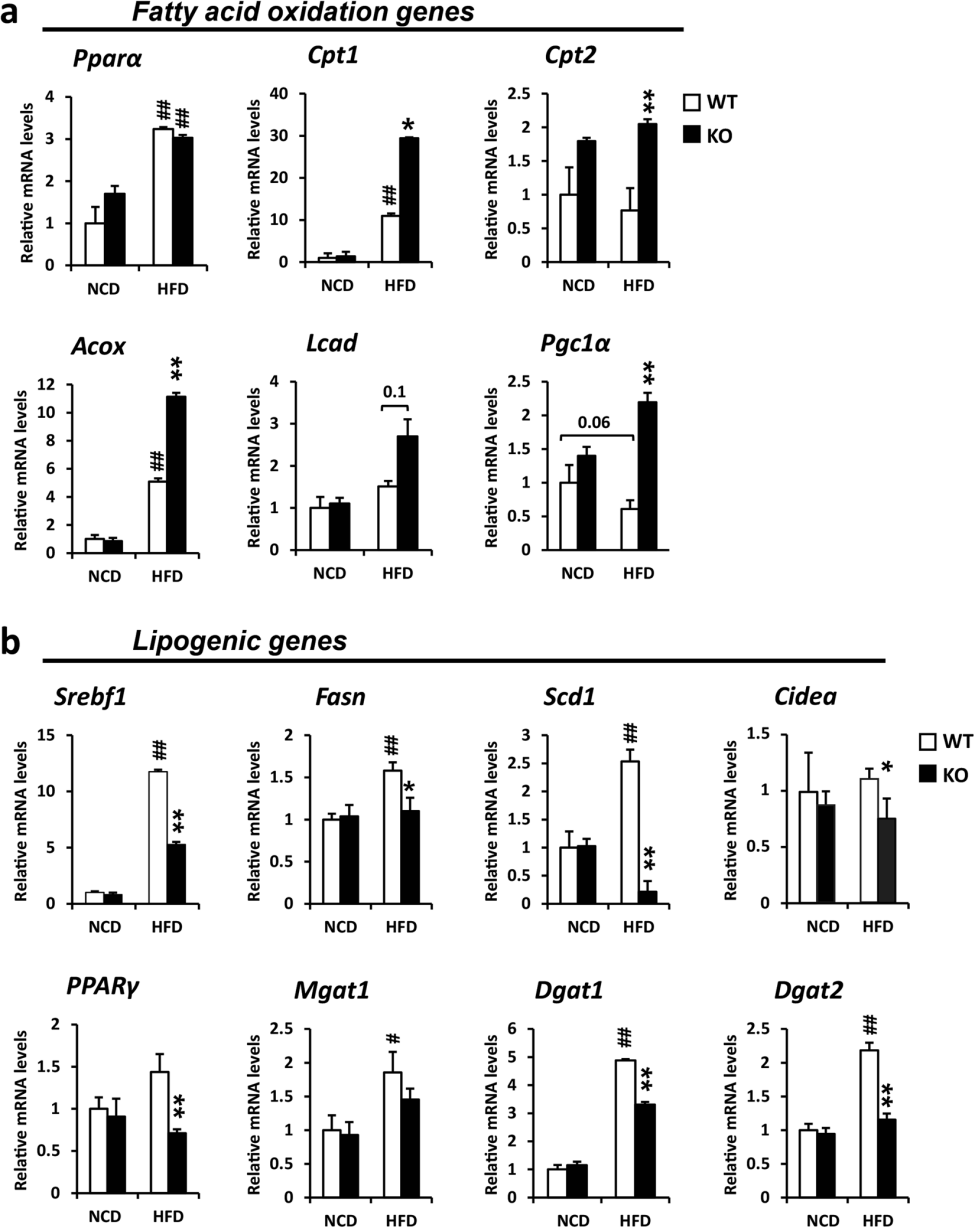
*Ahnak* KO mice show alterations in the expression of key genes involved in lipid metabolism. Relative mRNA expression of key genes involved in fatty acid oxidation (**a**) and lipogenesis (**b**) as measured by quantitative real-time PCR in the livers of *Ahnak* KO and WT mice fed chow and a HFD for 7 weeks. Data were presented as the means ± SEM. *p < 0.05; **p < 0.01, versus HFD-fed WT mice; ^#^ *p* < 0.05; ^##^ *p* < 0.01, versus NCD-fed WT mice.

FGF21 regulates hepatic fatty acid oxidation and ketogenesis^34^. To determine the functional consequence of FGF21 induction in *Ahnak* KO mice receiving a HFD, we measured the expression of key genes involved in lipid metabolism. The mRNA level of PPARα, a key regulator of fatty acid oxidation, was not altered in either NCD- or HFD-fed *Ahnak* KO mice compared with WT mice. However, PPARα target genes, including *Cpt1*, carnitine palmitoyltransferase 2 (*Cpt2*), acyl-coenzyme A oxidase 1 (*Acox*), acyl-coenzyme A dehydrogenase (*Lcad*), and *Pgc1α*, were found to be significantly increased in *Ahnak* KO mice compared with WT mice (Fig. 4a).

The lipogenic pathway in the liver has been determined to have been severely compromised during hepatic steatosis. Therefore, we examined the changes in lipogenic pathway-related gene expression. *Ahnak* KO mice that were fed a HFD showed inactivation of a hepatic lipogenic pathway that included the downregulation of genes required for fatty acid synthesis and TG esterification (Fig. 4b). We observed decreased expression of sterol regulatory element binding transcription factor 1c (*Srebf1*) mRNA, a key regulator of de novo lipogenesis, consistent with changes observed in the expression of its targets, including fatty acid synthase (*Fasn*) and stearoyl-coenzyme A desaturase 1 (*Scd1*). Hepatic mRNA expression of peroxisome proliferator-activated receptor gamma (*Pparγ*) and its targets, including diacylglycerol O-acyltransferase 1 (*Dgat1*) and diacylglycerol O-acyltransferase 2 (*Dgat2*), which catalyze the final step in TG esterification, was also decreased in HFD-fed *Ahnak* KO mice compared with HFD-fed WT mice. Additionally, the expression of cell death-inducing DNA fragmentation factor alpha subunit-like effector A (*Cidea*), a lipid droplet-associated protein that has emerged as an important regulator of lipid storage, and the formation of large lipid droplets in adipocytes and hepatocytes^35^, was also decreased in the livers of HFD-fed *Ahnak* KO mice compared with WT mice (Fig. 4b). These results indicate that a deficiency in the *Ahnak* gene in mice affects hepatic gene expression associated with the lipogenic pathway.

### HFD-fed *Ahnak* KO mice exhibit increased whole-body energy expenditure

**Fig. 5:**
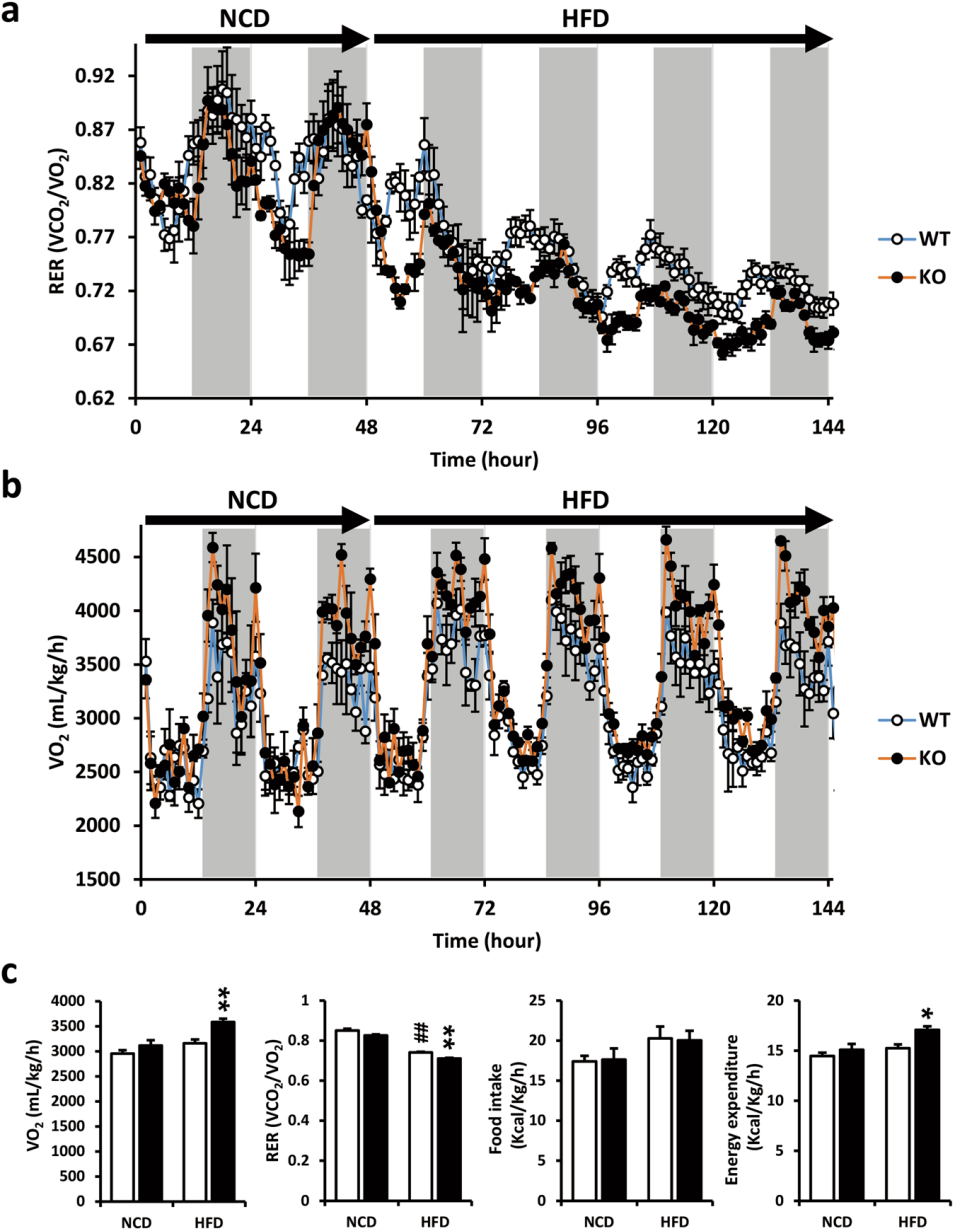
*Ahnak* KO mice have increased whole-body energy expenditure when fed a HFD. Metabolic parameters were determined using indirect calorimetry for *Ahnak* KO mice and wild-type littermates placed on a NCD followed by a HFD. **a** O_2_ consumption. **b** RER. **c** Quantitative values of VO_2_, RER, food intake, and energy expenditure. Data were presented as the means ± SEM (*n* = 5). *p < 0.05.

FGF21 has been identified to exert an effect on energy metabolism and body weight^8,9,36^. As shown in Fig. 1, compared with their WT littermates fed a HFD, *Ahnak* KO mice appeared to exhibit decreased fat mass^24^ accompanied by reduced liver weight. Furthermore, the production and circulation of FGF21 were increased in *Ahnak* KO mice receiving a HFD (Fig. 3a–c). Therefore, we determined whether *Ahnak* deficiency in mice affects energy metabolism through increased FGF21 expression. To assess whether the induction of FGF21 is responsible for energy consumption in *Ahnak* KO mice, calorimetry was assessed in *Ahnak* KO mice fed a NCD for 2 days, followed by a HFD for 4 days to induce FGF21 expression. As shown in Fig. 5a–c, *Ahnak* KO mice exhibited an increase in VO_2_ rates during HFD conditions throughout the light and dark cycles. Daily food intake was similar between WT and *Ahnak* KO mice for both the NCD and HFD. Comprehensive animal monitoring studies indicated that the rate of oxygen consumption (VO_2_) was increased in *Ahnak* KO mice. The calculated daily energy expenditure was also increased in *Ahnak* KO mice when switched to a HFD. Furthermore, the RER was reduced in *Ahnak* KO mice during the light and dark phases (Fig. 5c and Supplementary Fig. 5). This suggests that *Ahnak* KO mice prefer lipids as an energy source rather than carbohydrates compared with WT mice. Consistent with the augmented hepatic and circulating levels of FGF21 in HFD-fed *Ahnak* KO mice (Fig. 3b, c), *Ahnak* deficiency was associated with improved energy expenditure (Fig. 5), increased hepatic expression of genes involved in fatty acid oxidation (Fig. 4a), and reduced expression of genes that control lipogenesis (Fig. 4b).

### *Ahnak* deficiency attenuates palmitate-induced lipid accumulation in primary hepatocytes through FGF21 induction

**Fig. 6:**
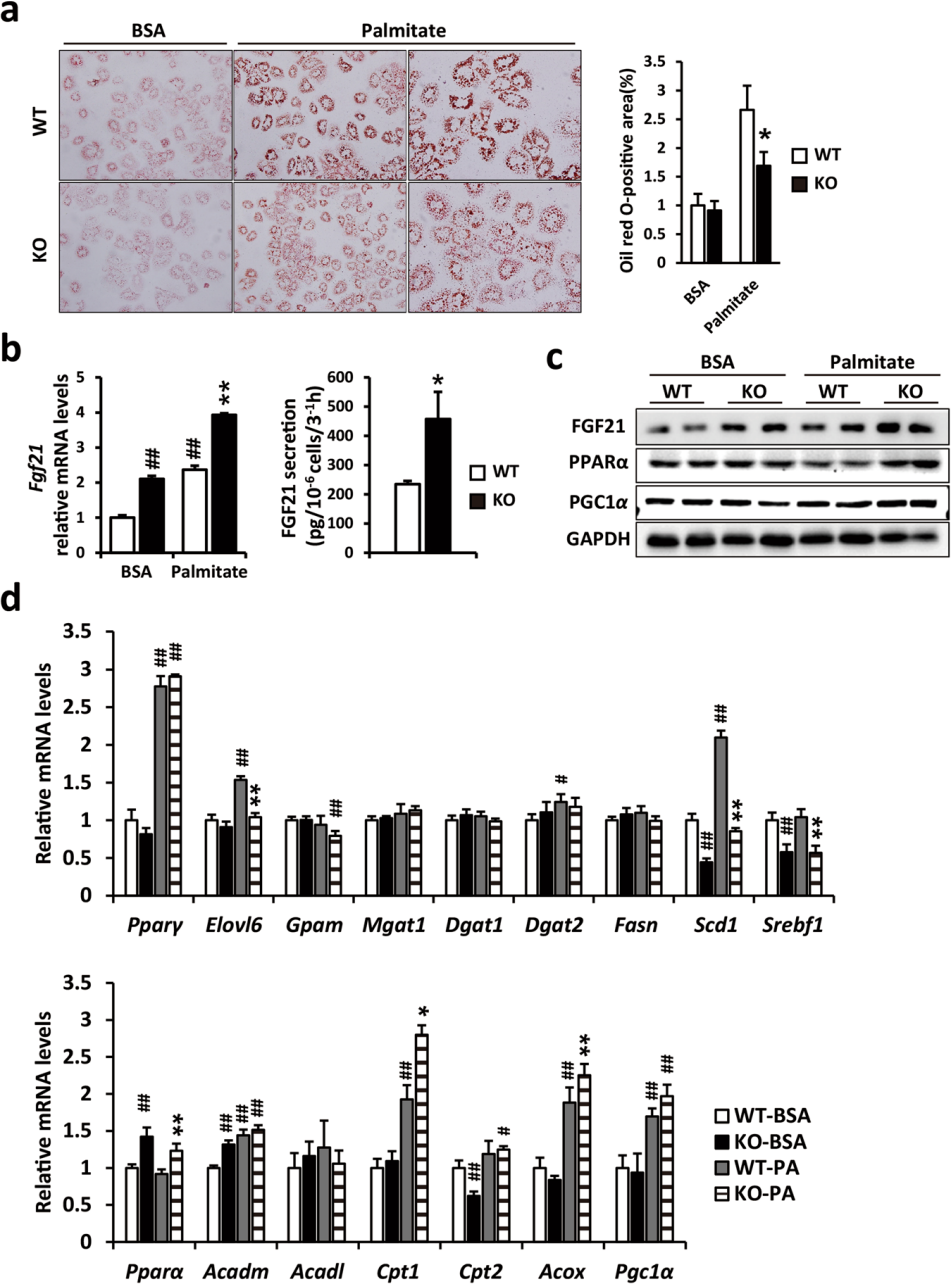
Loss of *Ahnak* in hepatocytes attenuates palmitate-induced lipid accumulation through FGF21 induction. **a** Primary hepatocytes were incubated with 0.25 mM palmitate for 24 h. Lipid accumulation was estimated by Oil Red O staining after palmitate incubation. Quantitation of the positive areas of Oil Red O staining is shown. **b** Relative FGF21 mRNA expression was measured after BSA and palmitate treatment (left). Secreted FGF21 was measured in culture media from WT and *Ahnak*-deficient hepatocytes (right). **c** Protein expression levels of FGF21, PPARα, and PGC1α were measured in WT and *Ahnak*-deficient hepatocytes incubated with 0.25 mM BSA or palmitate for 24 h. **d** Relative mRNA expression levels of genes involved in lipogenesis and fatty acid oxidation were measured after BSA and palmitate treatment. Data were presented as the means ± SEM. *p < 0.05; **p < 0.01, versus palmitate-treated WT hepatocytes; ^#^*p* < 0.05; ^##^*p* < 0.01, versus BSA-treated WT hepatocytes.

To further analyze the role of A*hnak* in lipid accumulation in hepatocytes, we examined palmitate-induced lipid accumulation in primary hepatocytes isolated from *Ahnak* KO and WT mice. Oil Red O staining showed that lipid droplets in palmitate-treated WT hepatocytes were higher than those in *Ahnak*-deficient hepatocytes (Fig. 6a).

We next examined whether *Ahnak* deficiency regulates both the production and secretion of FGF21 in primary hepatocytes. As shown in Fig. 6b, the expression of *Fgf21* was increased in *Ahnak*-deficient hepatocytes compared with WT hepatocytes and further increased by palmitate treatment of *Ahnak*-deficient hepatocytes (Fig. 6b). Consistent with *Fgf21* mRNA expression, FGF21 protein levels were also increased in palmitate-treated *Ahnak*-deficient hepatocytes compared with WT hepatocytes. A concomitant increase in the expression of PPARα protein was also observed in palmitate-treated hepatocytes isolated from *Ahnak* KO mice (Fig. 6c). To determine the level of FGF21 released from hepatocytes, we measured the concentration of FGF21 in the culture media obtained from *Ahnak*-deficient and WT hepatocytes. As expected, the level of secreted FGF21 was increased in *Ahnak*-deficient hepatocytes compared with WT hepatocytes (Fig. 6b).

To identify which molecular mechanism regulates lipid accumulation in *Ahnak*-deficient hepatocytes, the expression of lipid metabolism genes was measured (Fig. 6d). *Srebf1*, a key transcription factor regulating de novo lipogenesis, was significantly decreased in BSA- and palmitate-treated *Ahnak*-deficient hepatocytes compared with WT hepatocytes. *Scd1* expression was also significantly decreased in *Ahnak* KO hepatocytes. Furthermore, genes involved in fatty acid oxidation, such as *Acox*, *Cpt1*, *Cpt2*, and *PPARα*, were increased in *Ahnak* KO hepatocytes. These results indicate that palmitate-induced lipid accumulation was attenuated in *Ahnak*-deficient hepatocytes and may be further attributed to the induction of FGF21 and PPARα expression. Collectively, these data confirm that *Ahnak* deficiency contributes to the induction of the PPARα-FGF21 hormone axis in hepatocytes. This suggests that *Ahnak* deficiency improves hepatic steatosis largely through enhanced expression of FGF21 and genes associated with fatty acid oxidation.

## Discussion

In the present study, we examined the role of AHNAK in the regulation of hepatic lipid metabolism in diet-induced fatty liver. We found that *Ahnak* KO mice were resistant to diet-induced obesity and hepatic steatosis when fed a HFD. To identify the molecular mechanism underlying the protection from diet-induced hepatic steatosis, a microarray analysis was performed with liver samples from HFD-fed *Ahnak* KO and WT mice. The gene expression profiles revealed a similar cluster of genes in the liver of HFD-fed *Ahnak* KO mice compared with the NCD-fed WT and *Ahnak* KO mice. A gene ontology analysis of the downregulated DEGs (GSE70119) revealed that the oxidative reduction pathway, including fatty acid oxidation and inflammatory signaling pathways, was highly enriched in *Ahnak* KO mice. The expression of several genes involved in proinflammatory signaling was decreased in the *Ahnak* KO mice (Supplementary Fig. 3). The results indicate that excessive lipid accumulation in the liver may induce oxidative stress and the inflammatory response^37,38^. Thus, attenuated lipid accumulation in the liver of *Ahnak* KO mice fed a HFD may result in the downregulation of inflammatory signaling.

The most important finding of this study is the identification of FGF21 as a novel regulatory protein modulated by the genetic manipulation of *Ahnak*. Gene expression profiling revealed that *Fgf21* was increased by ~3.2-fold in the livers of *Ahnak* KO mice compared with WT mice fed a HFD. *Ahnak* deficiency in mice reportedly results in increased hepatic FGF21 production and release (Fig. 3a–c). It is well documented that FGF21 functions as a metabolic regulator in several diabetic animal models^39^. Administration of FGF21 can reduce hepatic TG accumulation and thus reverse body weight gain and hepatic steatosis in rodents and primates^12,13^. Additionally, FGF21 regulates energy homeostasis in adipocytes through an AMPK/SIRT1/PGC1α-dependent mechanism, resulting in enhanced oxidative metabolism and mitochondrial biogenesis^14,15^. Therefore, we focused on the role of FGF21 in preventing diet-induced hepatic steatosis in *Ahnak*-deficient mice. As expected, *Ahnak* KO mice exhibited an increase in hepatic expression and plasma levels of FGF21 compared with WT mice fed a HFD (Fig. 3).

We also found that *Ahnak* KO mice exhibited reduced expression of *Srebf1* (Fig. 4b) and its target genes, leading to the inhibition of hepatic lipid accumulation. Furthermore, *Ahnak* KO mice showed increased expression of genes involved in fatty acid oxidation through the PPARα/AMPK signaling pathway. Consistent with these results, the excessive lipid accumulation induced by palmitate treatment was prevented in *Ahnak*-deficient hepatocytes and *Ahnak* knockdown HepG2 cells, accompanied by increased expression of genes involved in fatty acid oxidation through the increased expression of PPARα protein and AMPK activation. Therefore, our study establishes that *Ahnak* deficiency in mice attenuates diet-induced hepatic lipid accumulation by activating PPARα/AMPK signaling. These findings also suggest that targeting AHNAK may have beneficial effects on the development of hepatic steatosis.

Hepatic expression of FGF21 is induced directly by PPARα in the liver in response to fasting and PPARα agonists^34,40,41^. The hepatic PPARα pathway plays a significant role in ketogenesis and fatty acid oxidation^42,43^. The mechanism is mediated, in part, by PPARα-dependent expression of Fgf21, a gene that is upregulated by *Ahnak* deficiency in mice. In the present study, we discovered that AHNAK modulates PPARα function, which is involved in fatty acid oxidation. *Ahnak* deficiency or deletion promotes fatty acid oxidation in the liver, contributing to the improvement in hepatic steatosis. However, PPARα mRNA and protein levels between *Ahnak* KO and WT mice were not consistent. Although the expression of *Pparα* mRNA was similar between HFD-fed WT and KO mice (Fig. 4a), hepatic PPARα protein expression was increased in *Ahnak* KO mice after HFD feeding or palmitate treatment compared with that of WT mice (Figs. 3c and 6c). This inconsistency may be explained by earlier observations showing that PPARα turnover can be regulated by the ubiquitin-proteasome system in a ligand-dependent manner^44^. In addition, it was recently reported that hepatic PPARα function is controlled by polyubiquitination and proteasome-mediated degradation through the coordinated actions of PAQR3 and HUWE1^45^. Therefore, the discrepancy observed between the expression of PPARα mRNA and protein in *Ahnak* KO and WT mice could be due to posttranslational modification of PPARα, which is regulated by AHNAK. Furthermore, AHNAK has been identified as a new substrate of the ubiquitin-protein ligase E3C (UBE3C) in the regulation of p53 activity^46^. However, the direct mechanism linking AHNAK to PPARα stability has yet to be identified.

Circulating FGF21 regulates energy homeostasis in adipocytes through an AMPK/SIRT1/PGC1α-dependent mechanism, resulting in enhanced mitochondrial oxidative capacity^14,15^. Phosphorylation of AMPK and PGC1α protein expression was increased in the livers of HFD-*Ahnak* KO mice compared with WT mice. These results indicate that pronounced obesity and hepatic steatosis are attenuated in *Ahnak* KO mice fed a HFD. This may be attributed, in part, to the induction of FGF21, fatty acid oxidation, and inhibition of lipogenic genes in the liver.

To further understand how *Ahnak* deficiency inhibits lipid accumulation in the liver, we analyzed the changes in the expression of lipogenic genes. We found that hepatic expression of several genes involved in fatty acid uptake and lipid accumulation was decreased in HFD-fed *Ahnak* KO mice (Fig. 4a). *Ahnak* KO mice also exhibited decreased lipid uptake, consistent with decreased *Cd36* expression and expression of the lipogenic genes *Srebf1*, *Fasn*, *Scd1*, *Dgat1*, and *Dgat2*. SREBF1 and PPARγ are well-known transcriptional regulators of lipogenic gene expression in the liver^47^. Therefore, *Ahnak* KO mice may inhibit hepatic lipid accumulation through regulation of lipogenic gene expression. Although HFD feeding increased *Srebf1* expression in *Ahnak* KO mice compared with NCD-fed *Ahnak* KO mice (Fig. 4b), the expression of *Srebf1* in primary hepatocytes of *Ahnak* KO mice was not changed when comparing the BSA and palmitate treatment groups (Fig. 6d). In addition, the expression patterns of target genes such as *Fasn* and *Scd1* differed in the liver tissue (Fig. 4b) and primary hepatocytes (Fig. 6d), respectively. Because *Srebf1* gene expression is regulated by itself, it is difficult to explain this phenomenon based on whether AHNAK affects the transcriptional regulatory roles of SREBF1 or regulates *Srebf1* gene expression. To activate SREBF1, cleavage of SREBFs by the site-2 protease (S2P) within a transmembrane segment or near the membrane surface after an initial cleavage by site-1 protease (S1P) should occur due to sterols under HFD conditions. Moreover, it is known that SREBF1-mediated de novo fatty acid synthesis in the liver is negatively regulated by the insulin-induced gene (INSIG) molecule^48^. It was recently reported that AMPK interacts with and mediates phosphorylation of INSIG, resulting in the inhibition of SREBF1 cleavage and processing and thus attenuating lipogenic gene expression^49^. In the present study, the phosphorylated active form of AMPK was increased in the livers of HFD-fed *Ahnak* KO mice (Fig. 3c). Further studies are needed to clarify whether AHNAK has a regulatory role through the AMPK response in activating SREBF1 during HFD conditions. In addition, because whole-body *Ahnak* KO mice were used in our study, it should be considered that decreased inflammatory signaling in *Ahnak* KO mice may result in attenuated lipid accumulation. This has been strongly supported by an earlier observation that *Ahnak* KO mice showed CD4 + T cell inactivation and decreased cytokine secretion^50^. Thus, attenuated lipid accumulation in the liver of *Ahnak* KO mice fed a HFD may result in the downregulation of inflammatory signaling.

It is well established that FGF21 has an effect on energy metabolism and body weight^8,9,36^. To directly assess whether the induction of FGF21 in *Ahnak* KO mice is responsible for energy fluctuations, indirect calorimetry was used to assess *Ahnak* KO mice placed on a NCD followed by a HFD. As shown in Fig. 5a–c, *Ahnak* KO mice exhibited increased VO_2_ rates under HFD conditions throughout the light and dark cycles but not under NCD conditions. Daily food intake was determined to be similar between WT and *Ahnak* KO mice for both the NCD and HFD. Comprehensive animal monitoring studies indicated that the rates of oxygen consumption (VO_2_) were increased in *Ahnak* KO mice. The calculated daily energy expenditure was also increased in *Ahnak* KO mice when switched to a HFD. Moreover, the RER was reduced in *Ahnak* KO mice during the light and dark phases (Fig. 5a–c and Supplementary Fig. 5). This may indicate that *Ahnak* KO mice use more lipids as an energy source than carbohydrates when compared with WT mice. Overall, the results in *Ahnak* KO mice were characterized by a decrease in fat mass, an increase in energy expenditure, and no significant alterations in food intake.

To better understand the effects of *Ahnak* deficiency at the cellular level, we examined lipid accumulation in primary hepatocytes following exposure to palmitate. *Ahnak* deficiency in hepatocytes resulted in decreased expression of lipogenic genes and increased expression of genes involved in fatty acid oxidation, resulting in significantly attenuated lipid accumulation in palmitate-treated cells (Fig. 6d). We next examined whether *Ahnak* deficiency regulates the production and secretion of FGF21 in primary hepatocytes. As shown in Fig. 6b, *Fgf21* expression was increased in *Ahnak*-deficient hepatocytes compared with WT hepatocytes, and it was further increased by palmitate treatment of *Ahnak*-deficient hepatocytes. Consistent with mRNA expression, FGF21 protein levels were also increased in palmitate-treated *Ahnak*-deficient hepatocytes compared with WT hepatocytes (Fig. 6c). However, no differences were observed between BSA-treated *Ahnak*-deficient and WT hepatocytes. Consistent with FGF21 expression, PPARα protein was significantly increased in palmitate-treated hepatocytes isolated from *Ahnak* KO mice (Fig. 6c). To determine the level of secreted FGF21 from hepatocytes, we measured the concentration of FGF21 in culture media obtained from *Ahnak*-deficient and WT hepatocytes. The concentration of FGF21 was increased in serum-starved *Ahnak*-deficient hepatocytes compared with WT hepatocytes (Fig. 6b). These results indicate that palmitate-induced lipid accumulation was attenuated in *Ahnak*-deficient hepatocytes and can be attributed, in part, to the induction of FGF21 and PPARα expression in hepatocytes. These results are consistent with the observed decreased lipid accumulation in *Ahnak*-deficient hepatocytes treated with palmitate.

**Fig. 7:**
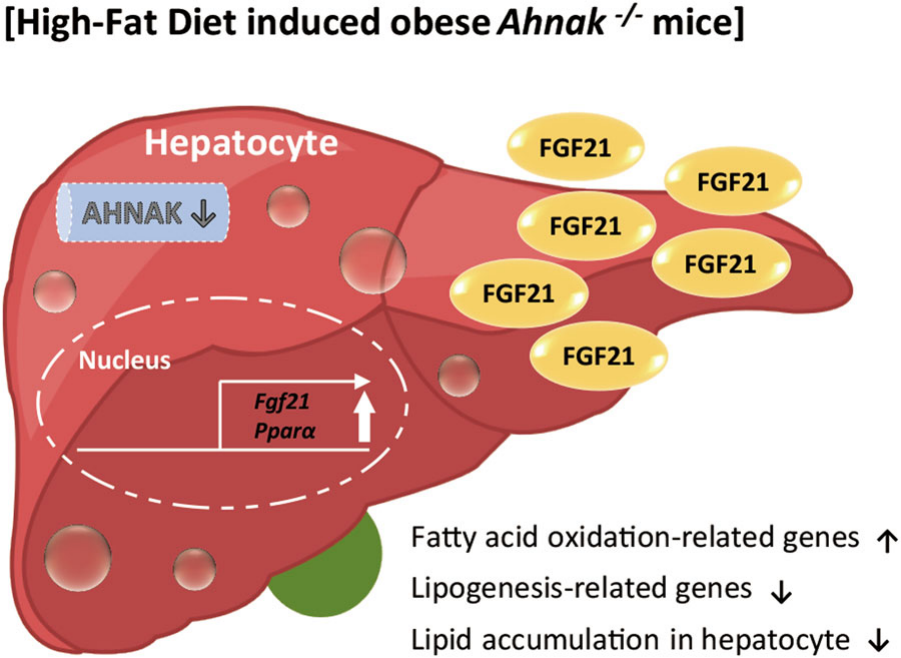
Summary of the effect of *Ahnak* deficiency on liver metabolism. High-fat diet-induced obese *Ahnak*-deficient mice exhibited increased hepatic FGF21 production and release compared with WT HFD-fed mice, thereby inducing the expression of genes involved in the fatty acid oxidation pathway, decreasing lipogenesis, decreasing hepatic steatosis, and increasing energy expenditure.

Characterization of the phenotypic changes in *Ahnak* KO mice, including reduced hepatic steatosis, decreased adiposity, and increased energy expenditure, establishes AHNAK as a novel regulator of FGF21 for the modulation of hepatic fatty acid metabolism. *Ahnak* deficiency in mice results in increased hepatic FGF21 production and release, reduced lipogenesis, and increased expression of genes involved in the fatty acid oxidation pathway (Fig. 7). The discovery of FGF21 induction in *Ahnak* KO mice is also supported by the fact that the production and secretion of FGF21 can be increased by *Ahnak* knockdown in hepatocytes. Therefore, our study establishes the role of AHNAK in regulating hepatocyte lipid metabolism through the PPARα/FGF21 axis. *Ahnak* deficiency has been reported to suppress lipid accumulation in the liver and may represent a novel therapeutic target to reduce the formation of fatty liver.

## Supplementary information

Supplementary information

## Data Availability

Experimental materials used to generate the results reported in this manuscript are available upon request.
