# Supplementary material for: Ahnak deficiency attenuates high-fat diet-induced fatty liver in mice through FGF21 induction
Source: Exp Mol Med. 2021 Mar 30;53(3):468–82. doi: 10.1038/s12276-021-00573-3 (PMC8080712; doi:10.1038/s12276-021-00573-3)
Supplement: Supplementary file 1 — Supplementary information [file 12276_2021_573_MOESM1_ESM.docx]

***Ahnak* deficiency attenuates high-fat diet-induced fatty liver in mice through FGF21 induction**

(Running title: *Ahnak* regulates hepatic lipid metabolism)

Yo Na Kim^1,2^, Jae Hoon Shin^1,2^, Dong Soo Kyeong^1,2,3^, Soo Young Cho^4^, Mi-Young Kim^2^, Hee Jung Lim^1,2,6^, Maria Raquel Rojas Jimenez^1,2^, Il Yong Kim^1,2^, Mi-Ock Lee^5^, Yun Soo Bae^6^, Je Kyung Seong^1,2,3^

**Contents**

1. Supplementary table
2. Supplementary figures
3. **Supplementary Table**

Supplementary Table 1. Sequence of Primers Used for Real-Time Quantitative PCR.

| Symbol | Primer sequence (5' to 3') |
| --- | --- |
| *Acadm* | GCAGCTGGCGCTGTCGGGCT |
|  | CGGCGTCAGTGGCTAGCTGATTG |
| *Acox1* | CCTGATTCAGCAAGGTAGGG |
|  | TCGCAGACCCTGAAGAAATC |
| *Acsl1* | ACCATCAGTGGTACCCGCTA |
|  | CGCTCACCACCTTCTGGTAT |
| *Ahnak1* | GCCAAAGGAAACAAACACC |
|  | AAATAAGCCCACATCCCATC |
| *Ahnak2* | ATGTCAAAGCAAAAGGCTTG |
|  | GTGTCCCACAGAGGATTCAG |
| *ApoB100* | CGTGGGCTCCAGCATTCTA |
|  | CCAATGGTCGGGCACTGCT |
| *Ccl2* | ATTGGGATCATCTTGCTGGT |
|  | CCTGCTGTTCACAGTTGCC |
| *Ccl3* | ACTGCCTGCTTCTCCTACA |
|  | AGGAAAATGACACCTGGCTGG |
| *Cd36* | ATTGGTCAAGCCAGCT |
|  | TGTAGGCTCATCCACTAC |
| *Cd68* | CTTCCCACAGGCAGCACAG |
|  | AATGATGAGAGGCAGCAAGAGG |
| *Cpt1a* | AGTGGCCTCACAGACTCCAG |
|  | GCCCATGTTGTACAGCTTCC |
| *Dgat1* | TCACCACACACCAATTCAGG |
|  | GACGGCTACTGGGATCTGA |
| *Dgat2* | TCTCAGCCCTCCAAGACATC |
|  | GCCAGCCAGGTGAAGTAGAG |
| *Elovl6* | GGAGCAGAGGCGCAGAGAAC |
|  | GAGCGGCTTCCGAAGTTCAA |
| *F4/80* | CTTTGGCTATGGGCTTCCAGTC |
|  | GCAAGGAGGACAGAGTTTATCGTG |
| *Fabp1* | CCCGAGGACCTCATCCAGAA |
|  | CCCCAGGGTGAACTCATTGC |
| *Fabp4* | TTCGATGAAATCACCGCAGA |
|  | AGGGCCCCGCCATCT |
| *Fasn* | GCTGCGGAAACTTCAGGAAAT |
|  | AGAGACGTGTCACTCCTGGACTT |
| *Gpam* | GCCAGCAAGTCCTGCGCTAT |
|  | CCTGCTCGTGTGGGTGATTG |
| *Hmgcs2* | ATACCACCAACGCCTGTTATG |
|  | CAATGTCACCACAGACCACCA |
| *Il6* | ACCAGAGGAAATTTTCAATAGGCT |
|  | GATGCACTTGCAGAAAACA |
| *Mcp1* | CTTCTGGGCCTGCTGTTCA |
|  | CCAGCCTACTTGGGATCA |
| *Mgat1* | CCAGCGCAAAGGGTTTGTT |
|  | CACCAAAAGAAAATACTGGAACCA |
| *Mttp* | GACCACCCTGGATCTCCATA |
|  | AGCGTGGTGAAAGGGCTTAT |
| *Pgc1α* | AGCCGTGACCACTGACAACGA |
|  | GCTGCATGGTTCTGAGTGCTA |
| *Pparγ1* | TCTTCCATCACGGAGAGGTC |
|  | GATGCACTGCCTATGAGCAC |
| *Pparγ2* | CCAGAGCATGGTGCCTTCGCT |
|  | CAGCAACCATTGGGTCAGCTC |
| *Pparα* | CAGTGGGGAGAGAGGACAGA |
|  | AGTTCGGGAACAAGACGTTG |
| *Tnfα* | ATGGCGTTTCCGAATTCAC |
|  | GAGGCAACCTGACCACTCTC |
| *36B4* | GAGGAATCAGATGAGGATATGGGA |
|  | AAGCAGGCTGACTTGGTTGC |

**2. Supplementary Figures**

**
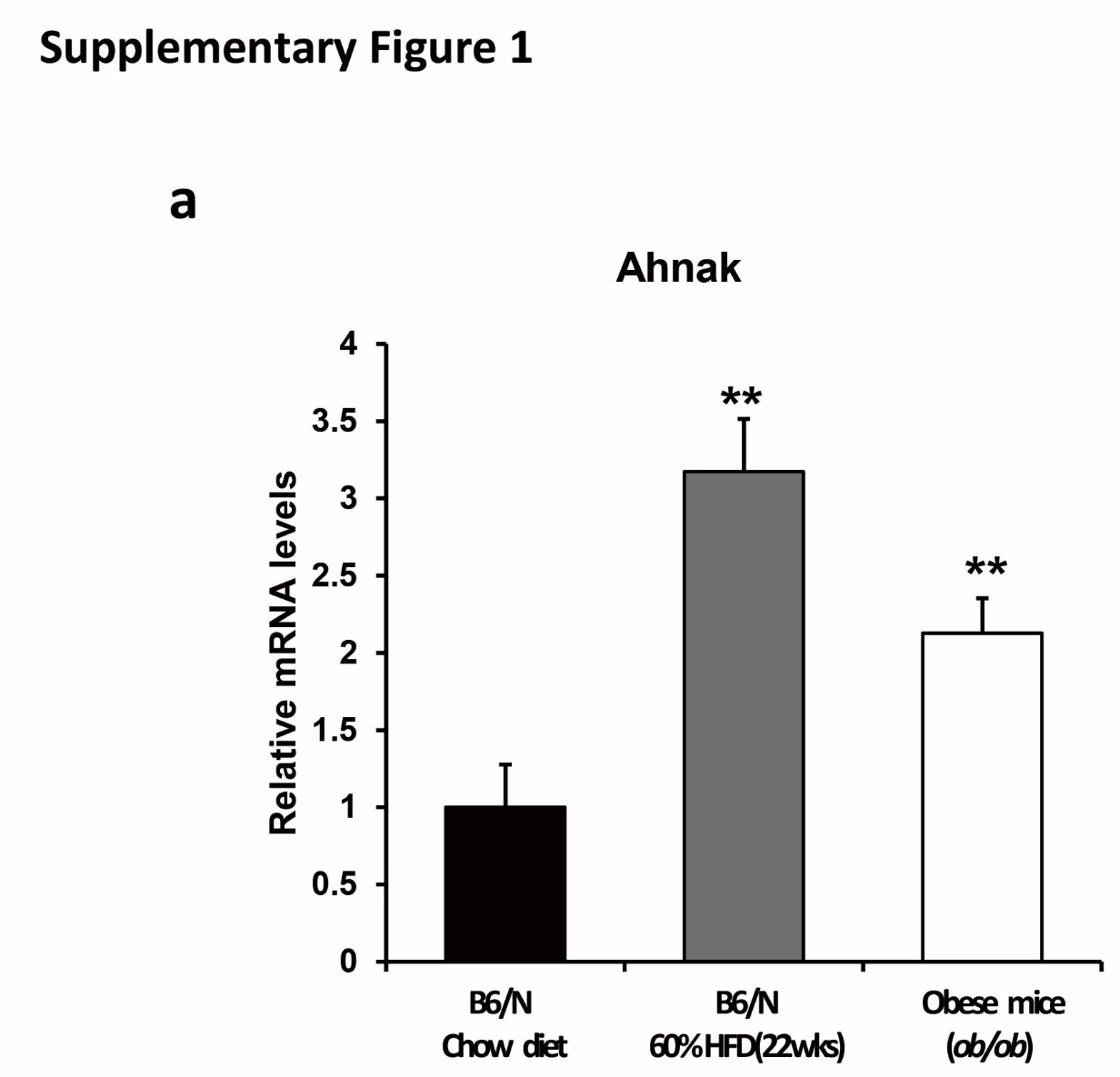
**

**Supplementary Figure 1. *Ahnak* mRNA expression increases in the liver of diet-induced and genetically obese mice.**

Relative mRNA expression of *Ahnak* was measured using quantitative real-time PCR in the liver of DIO and genetically obese mice.

**
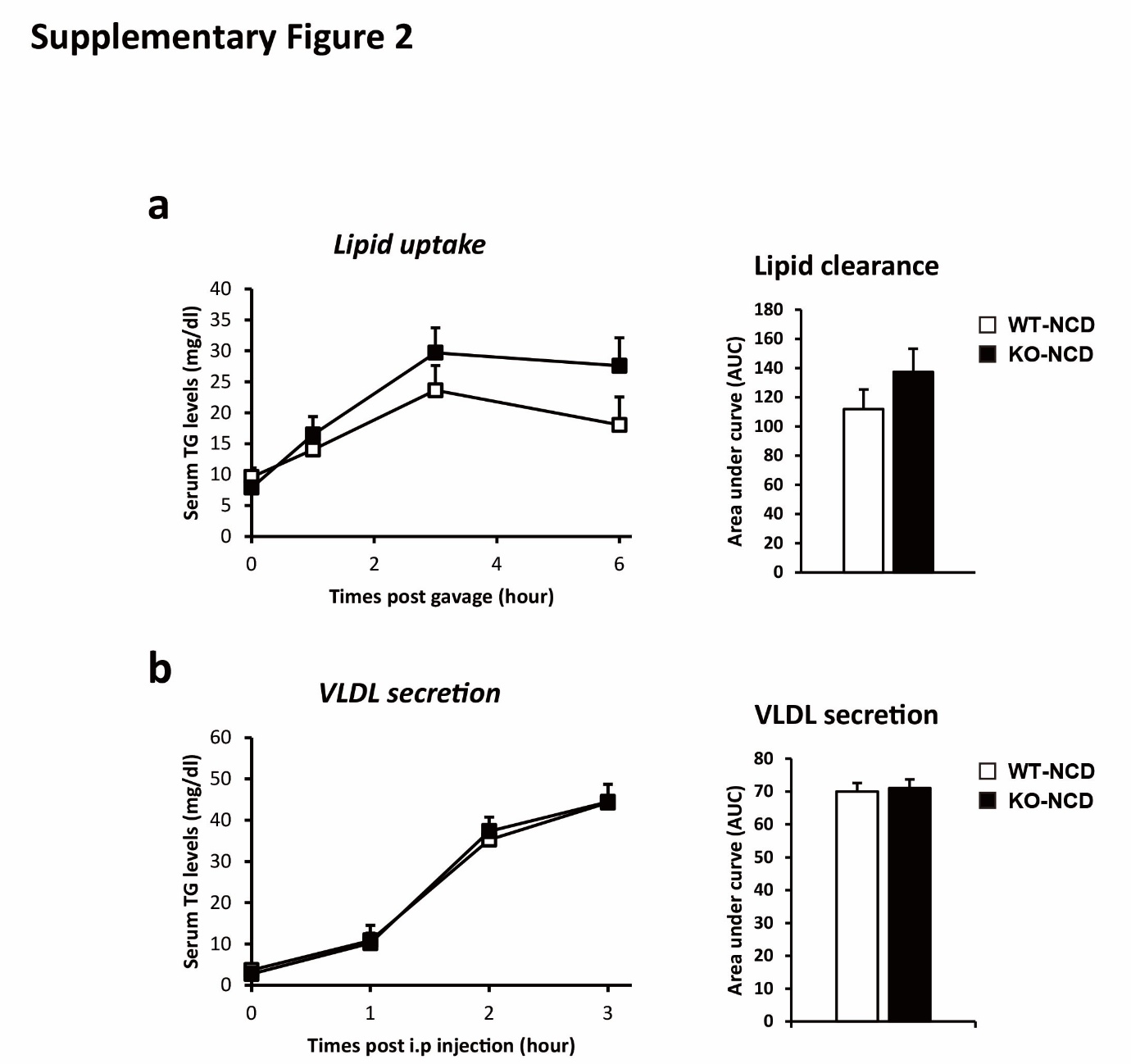
**

**Supplementary Figure 2. Measurement of lipid clearance and VLDL secretion in NCD-fed Ahnak-deficient mice**

(a) Lipid clearance was estimated by measuring blood TG levels after oral olive oil administration. (b) VLDL secretion. Mice were treated with Poloxamer 407 in order to block VLDL clearance, and VLDL secretion was estimated by measuring blood TG levels after Poloxamer 407 treatment. Data are presented as the means ±SEM. * p< 0.05; ** p<0.01.

**
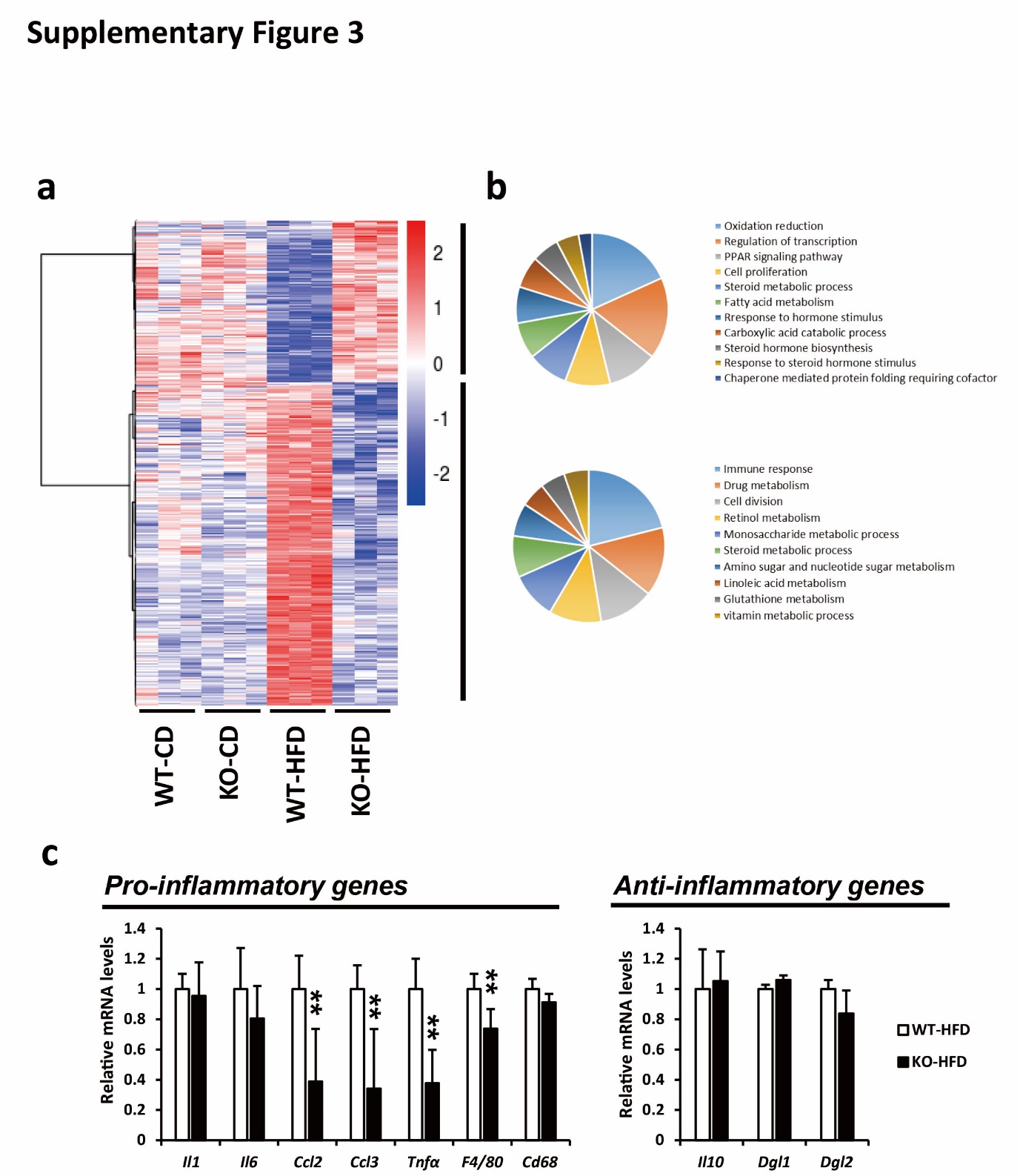
**

**Supplementary Figure 3. Differential gene expression that is observed between the livers of NCD- and HFD-fed WT and *Ahnak* KO mice as determined by microarray analysis.**

(a) Hierarchical clustering of samples using Ward with average linkage. Dendrogram and heat maps show replicate group similarity with differential and reproducible gene expression patterns between each group. (b) Ontology diagrams from up- and downregulated differential expression gene (DEG) greater than 1.5-fold in the liver of HFD-fed *Ahnak* KO mice compared with WT mice. The number of genes in each ontology category has been indicated. (c) Relative mRNA expression of hepatic genes involved in the inflammation response was measured using quantitative real-time PCR. Data are presented as the means ±SEM. * p < 0.05; ** p < 0.01.

**
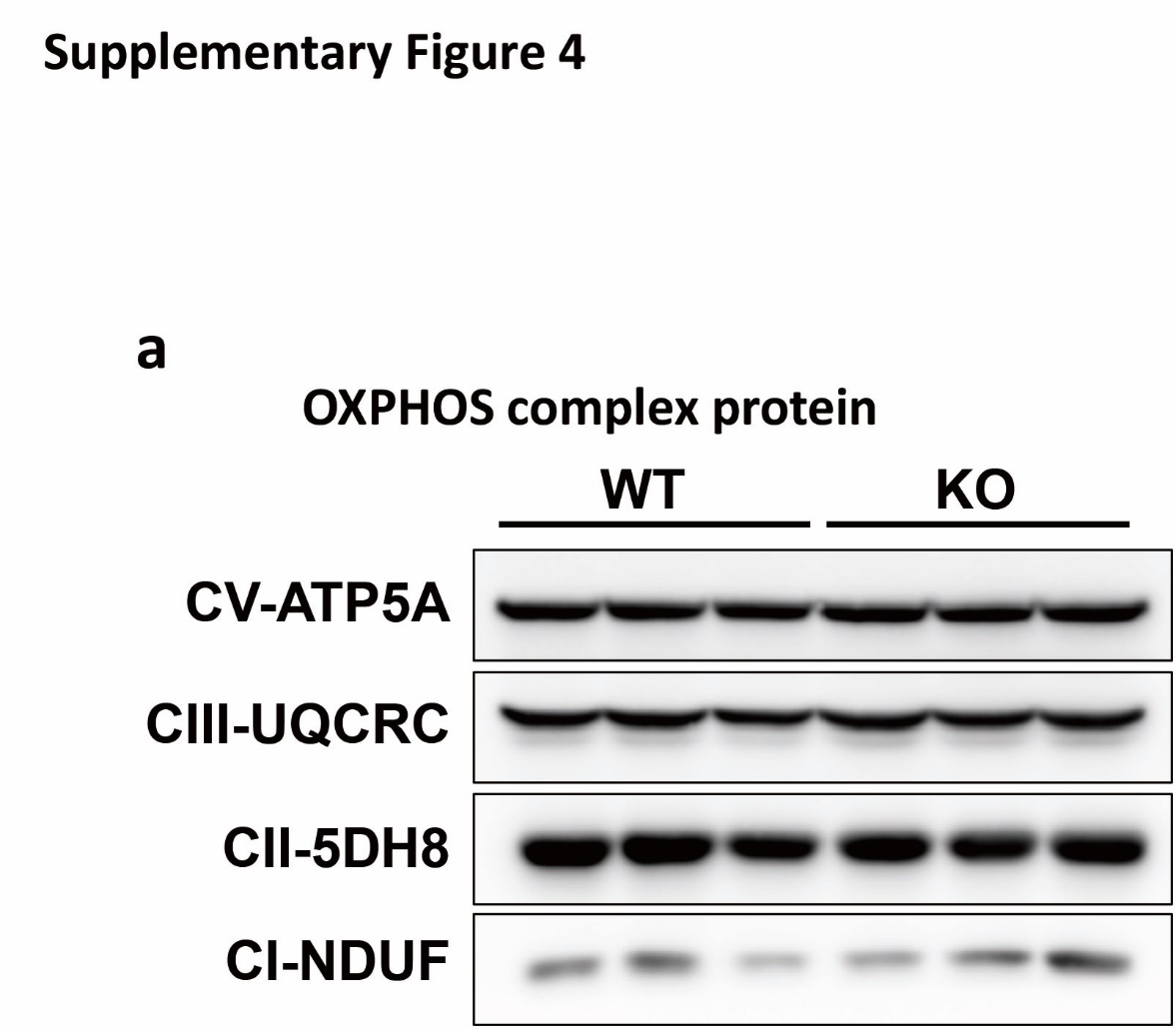
**

**Supplementary Figure 4. Immunoblots of OXPHOS complex proteins**

(a) Immunoblots for protein expression involved in the OXPHOS complex.

**
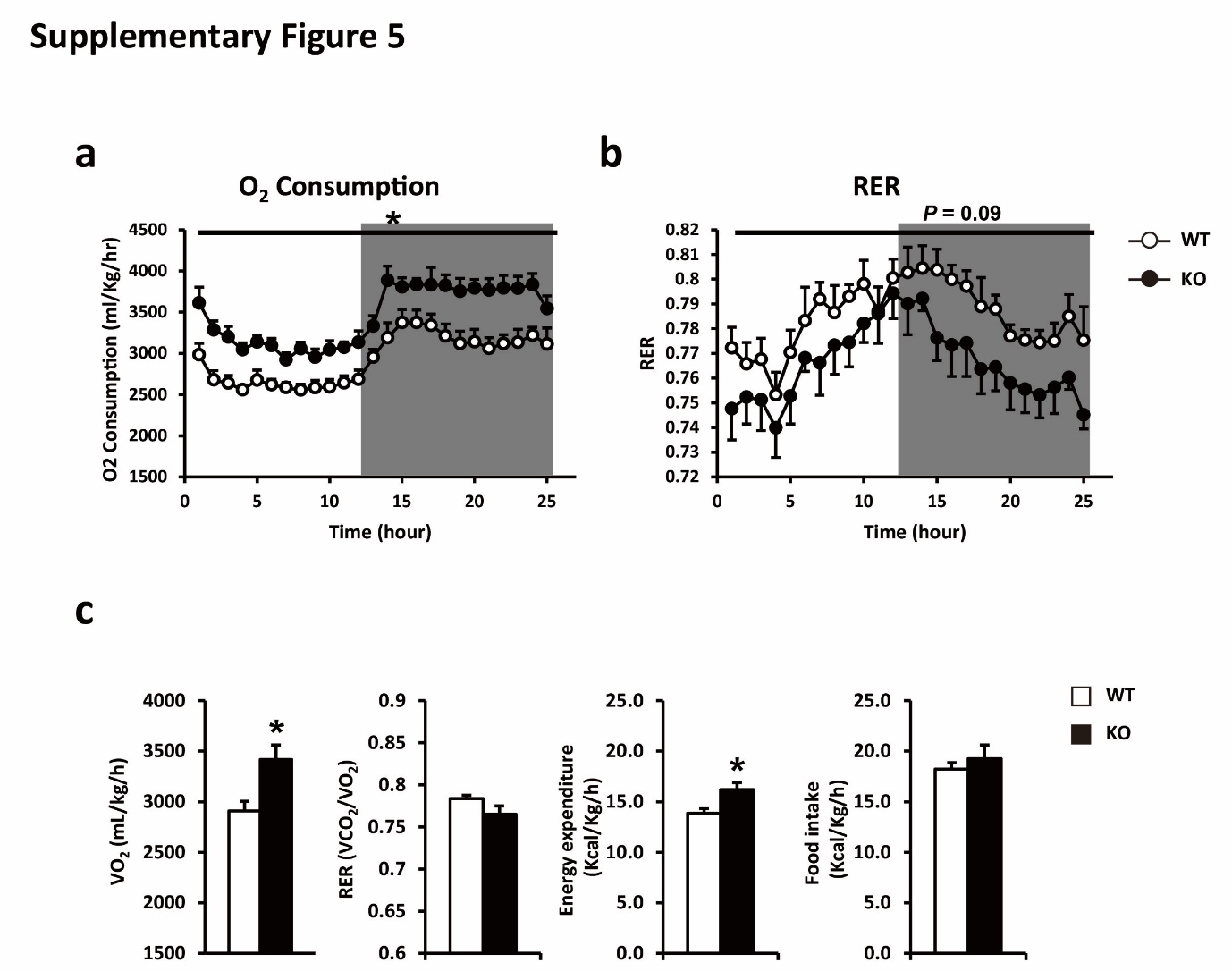
**

**Supplementary Figure 5. Whole-body energy expenditure increases in HFD-fed *Ahnak* KO mice.**

Metabolic parameters were determined using indirect calorimetry with *Ahnak* KO mice corresponding to wild-type littermates which were fed an HFD for 8 weeks. (a) O_2_ consumption, (b) energy expenditure, and (c) quantitative values of VO_2_, RER, energy expenditure, and food intake. Data are presented as the means ±SEM (n = 5). * p < 0.05
